# High Abundance of the Epibenthic Trachymedusa *Ptychogastria polaris* Allman, 1878 (Hydrozoa, Trachylina) in Subpolar Fjords along the West Antarctic Peninsula

**DOI:** 10.1371/journal.pone.0168648

**Published:** 2017-01-04

**Authors:** Laura J. Grange, Craig R. Smith, Dhugal J. Lindsay, Bastian Bentlage, Marsh J. Youngbluth

**Affiliations:** 1 Department of Oceanography, University of Hawai’i at Mānoa, Honolulu, Hawaii, United States of America; 2 Japan Agency of Marine-Earth Science and Technology (JAMSTEC), Natsushima-cho, Yokosuka, Kanagawa, Japan; 3 Department of Invertebrate Zoology, Smithsonian Institution, National Museum of Natural History, Washington DC, United States of America; 4 Harbor Branch Oceanographic Institute, Florida Atlantic University, Fort Pierce, Florida, United States of America; Auckland University of Technology, NEW ZEALAND

## Abstract

Medusae can be conspicuous and abundant members of seafloor communities in deep-sea benthic boundary layers. The epibenthic trachymedusa, *Ptychogastria polaris* Allman, 1878 (Hydrozoa: Trachylina: Ptychogastriidae) occurs in the cold, high latitude systems of both the northern and southern hemispheres, with a circumpolar distribution in Arctic and sub-Arctic areas, and disjunct reports of a few individuals from Antarctica. In January-February 2010, during benthic megafaunal photosurveys in three subpolar fjords along the West Antarctic Peninsula (Andvord, Flandres and Barilari Bays), *P*. *polaris* was recorded in Antarctic Peninsula waters. The trachymedusa, identified from megacore-collected specimens, was a common component of the epifauna in the sediment floored basins at 436–725 m depths in Andvord and Flandres Bays, reaching densities up to 13 m^-2^, with mean densities in individual basins ranging from 0.06 to 4.19 m^-2^. These densities are 2 to 400-fold higher than previously reported for *P*. *polaris* in either the Arctic or Antarctic. This trachymedusa had an aggregated distribution, occurring frequently in Andvord Bay, but was often solitary in Flandres Bay, with a distribution not significantly different from random. Epibenthic individuals were similar in size, typically measuring 15–25 mm in bell diameter. A morphologically similar trachymedusa, presumably the same species, was also observed in the water column near the bottom in all three fjords. This benthopelagic form attained abundances of up to 7 m^-2^ of seafloor; however, most *P*. *polaris* (~ 80%), were observed on soft sediments. Our findings indicate that fjords provide a prime habitat for the development of dense populations of *P*. *polaris*, potentially resulting from high and varied food inputs to the fjord floors. Because *P*. *polaris* resides in the water column and at the seafloor, large *P*. *polaris* populations may contribute significantly to pelagic-benthic coupling in the WAP fjord ecosystems.

## Introduction

Pelagic organisms, particularly medusae, are common members of benthic boundary layer communities, where some species may shift to benthic life stages, scavenge epibenthic food sources and attain appreciable seafloor abundance (e.g. [1, 2, 3, 4, 5, 6]). Despite a paucity of samples, the existence of a variety of gelatinous fauna in the benthic boundary layer is generally accepted [7]. The diversity and abundance have been attributed to increased prey availability in the form of detritivores that feed on marine snow sinking from the euphotic zone and organic material resuspended from the seafloor [2, 8, 9].

The fragility of gelatinous zooplankton, and absence of suitable sampling gear and preservatives, have hindered effective collection of intact specimens and contributed to their poor representation in deep-sea faunal inventories (summarised in [10]). In the last two decades imaging surveys and *in situ* collections, using underwater optical and acoustical instruments (summarised by [11]), towed underwater cameras, manned and remotely operated submersibles and autonomous underwater vehicles documented the occurrence and activities of gelatinous animals living near or on the seafloor [6, 12, 13, 14]. In addition, plankton nets tows [15] and sediment traps [16, 17], as well as gear adapted for dedicated sampling of medusae (e.g. nets on epibenthic sleds and bottom trawls as described in [18]), have facilitated collection of gelatinous organisms from deep benthopelagic habitats.

Despite these recent advances and extensive records of Hydrozoa from the early 1800’s onward [18, 19], benthopelagic gelatinous zooplankton in polar seas are poorly acknowledged, particularly when compared to crustaceans, such as euphausiids and copepods [20, 21, 22, 23]. In most cases high latitude studies of gelatinous zooplankton have been limited to species descriptions [18, 24, 25, 26]. Recent investigations have provided data on abundance and distribution records [21, 23, 27]. Most of these descriptions have been based on classical taxonomic approaches [28, 29, 30]. This study is one of few presenting a molecular analysis and DNA barcoding to validate species identifications.

The trachymedusa *Ptychogastria polaris* Allman, 1878 [31], is a cold-water species, occurring at high latitudes in the northern and southern hemispheres [25, 26, 32, 33, 34]. Allman described *P*. *polaris* from a single specimen collected off East Greenland (81’44°N, 64’45°W) [31]. Subsequent reports have recognised three species in the genus *Ptychogastria*: the Arctic *Ptychogastria polaris*, the Antarctic *Ptychogastria opposita* Vanhöffen, 1912 [35], and a Mediterranean counterpart *Ptychogastria asteroides* Haeckel, 1879 [36]. A further species, *Ptychogastria antarctica* Haeckel, 1879, was described as *Pectis antarctica* Haeckel, 1879 [36] but subsequently moved into the genus *Ptychogastria* by [37], where it remained as a doubtful species until recently being found to be conspecific with *Voragonema laciniata* Bouillon, Pagès & Gili, 2001 [18], which therefore becomes a junior synonym of *Pe*. *antarctica* [38]. *Ptychogastria asteroides* is the smallest member of the genus and considered to be a true *Ptychogastria* [33]. *Ptychogastria opposita* has been identified as congeneric and a true Antarctic representative of *Ptychogastria*; however a lack of distinguishing features led to the combination of *P*. *polaris* and *P*. *opposita* into a single bipolar species [33, 34]. This trachymedusa has a circumpolar distribution in the Arctic and subarctic [24, 25, 26, 32, 39, 40, 41], with specimens occasionally collected in deep-shelf waters of the temperate Atlantic and Pacific Oceans, including the Strait of Georgia (200–580 m; British Columbia), Kurile Islands (200 m; NW Pacific Ocean), Japan Sea (461 m) and Monterey Canyon (350–1000 m; California) [2, 26, 42, 43]. Arctic *P*. *polaris* are patchily distributed on the seafloor, with abundances between 0.01 and 0.91 m^-2^ in NE Greenland and 0.01 and 0.76 m^-2^ in the Barents Sea [25, 26]. In Antarctic waters, most records of *P*. *polaris* are disjunct, with a few specimens collected from two widely separated areas near Gauss Station (66’02°S, 90’20°E) and the South Shetland Islands (61–63°S, 53–61°W) [33, 35]. Recently, this trachymedusa was reported as one of the numerically dominant epibenthic megafaunal species in two West Antarctic Peninsula (WAP) fjords [44]. *Ptychogastria polaris* is therefore one of 23 bipolar species of Medusozoa [45], although the occurrences listed above suggest this trachymedusa may in fact be cosmopolitan–at least in cold, deep waters.

Here we describe patterns of abundance, distribution, body size and environmental conditions for *P*. *polaris*, and provide morphological and molecular comparisons to document the species’ phylogeny. We hypothesise that this trachymedusa may contribute significantly to pelagic-benthic coupling in WAP fjord-floor communities.

## Materials and Methods

### Morphological taxonomy and molecular systematics

**Fig 1:**
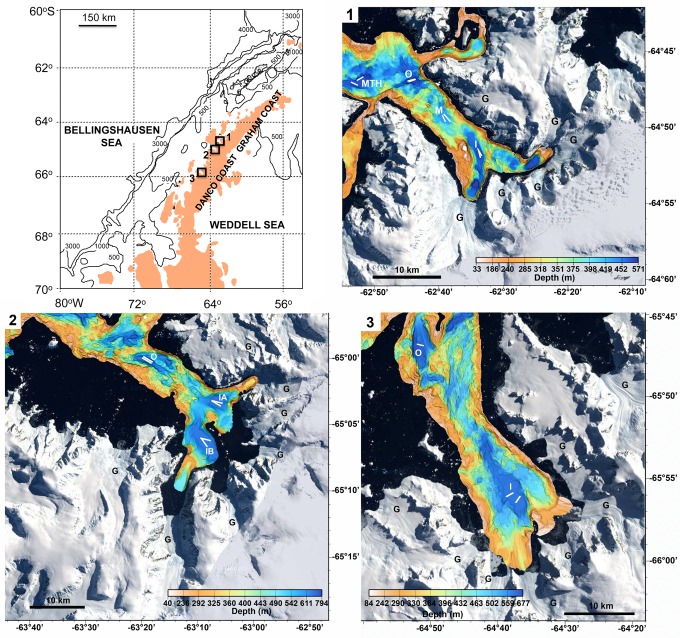
Distribution of sampling sites in subpolar fjords. Boxes indicate the subpolar WAP fjords (**1**) Andvord, (**2**) Flandres and (**3**) Barilari Bays. Panels 1–3: multibeam bathymetry superimposed on satellite imagery of the three WAP fjords. White lines indicate phototransect positions: I = inner basin (IA = inner basin A and IB = inner basin B); M = middle basin; O = outer basin; and MTH = fjord mouth. ‘G’ indicates the location of a tidewater glacier. Note that each fjord has multiple tidewater glaciers 10–15 km long carrying ice from the Peninsula ice cap (previously described by [46]). Data available from the U.S. Geological Survey. Satellite images are public domain USGS Products. Reprinted from [44].

Live individuals of *Ptychogastria polaris* were collected in Andvord and Flandres Bays (Fig 1), from the RVIB *Nathaniel B*. *Palmer* (cruise NBP10-01) and the ARSV *Lawrence M*. *Gould* (cruise LMG11-05) in February 2010 and May 2011, respectively (Table 1). These individuals were obtained from the top water of megacores (10 cm diameter) (OSIL Environmental Instruments and Systems) from which formalin-preserved (3) and frozen at -80°C (4) voucher specimens were saved for taxonomic identification. All collections were made in international waters, under the auspices of, and with permission from, the United States Antarctic program (USAP). No endangered or protected species were collected in this study. Megacore-collected trachymedusae were humanely sacrificed by rapid freezing, or by rapid warming to room temperature (which anesthetizes Antarctic marine benthos adapted to living at -1.0°C). Field collections of invertebrates within the USAP do not require IACUC approval.

**Table 1:** Station locations and environmental CTD data for megacore-collected specimens of *Ptychogastria polaris* from Andvord and Flandres Bays.

Sampling date	Cruise	Station (fjord basin)	Megacore (CRS & Tube #)	Preservation method	Latitude	Longitude	Mean depth (m)	Temp-erature (°C)	SalinityPSU	Dissolved oxygen (ml/L)
20/02/2010	NBP10-01	AO	1339 **#**11	Formalin 10%	-64.77934	-62.72885	556	0.15	34.50	5.55
20/02/2010	NBP10-01	AO	1339 **#**3	Formalin 10%	-64.77934	-62.72885	556	0.15	34.50	5.55
15/05/2011	LMG11-05	FIA	1351 **#**4	Formalin 10%	-65.05712	-63.12767	690	1.15	34.55	-
16/05/2011	LMG11-05	FIA	1355 #7	-80°C	-65.05383	-63.10942	701	1.15	34.55	-
27/05/2011	LMG11-05	AMTH	1369 #5	-80°C	-64.78498	-62.88118	533	0.00	34.51	5.56
27/05/2011	LMG11-05	AMTH	1369 #8	-80°C	-64.78498	-62.88118	533	0.00	34.51	5.56
27/05/2011	LMG11-05	AI	1372 #5	-80°C	-64.86145	-62.56255	539	0.26	34.53	-

Station locations and environmental CTD data for morphological (formalin-preserved) and molecular, megacore-collected specimens sampled during NBP10-01 (February 2010) and LMG11-05 (May 2011) from RVIB *Nathaniel B*. *Palmer* and ASRV *Lawrence M*. *Gould*, respectively. Fjord basins are indicated as follows: AMTH = Andvord Bay mouth; AO = Andvord Bay outer; AI = Andvord Bay inner; and FIA = Flandres Bay inner A. N.B. Dashes indicate that data are not available.

Formalin-preserved trachymedusae (Table 1) were compared to three voucher specimens of the subboreal *P*. *polaris* obtained from JAMSTEC collections: 1 specimen (5% formalin), JAMSTEC No. 045607 (2K1284SS7c), collected from Shiribeshi Seamount, Sea of Japan (43.46' N, 139.54' E), 234 m, 19 July 2001 (Cruise NT01-07 Leg 2, Dive no. 2K#1284) [4.9°C, salinity 34.31, dissolved oxygen 1.8 ml/L]; and 2 specimens (3% formalin), JAMSTEC No. 1120031607, 1120031609 (7K549SS5, 7K549SS6), collected off Okushiri Island, Sea of Japan (42.30' N, 139.47' E), 1062 m, 10 March 2012 (Cruise KR12-07, Dive no. 7K#0549) [0.24°C, salinity 34.01]. All formalin-preserved specimens were examined under a Leica MZ16 dissecting microscope with a Leica KL2500LCD illuminator under transmitted, darkfield and polarized light conditions.

For DNA sequencing, tentacle tissue was taken from each of the four frozen specimens and transferred immediately into -20°C molecular-grade (99.5%) EtOH. DNA was extracted from tissues using the Biosprint 96 workstation (Qiagen, Hilden, Germany) in conjunction with the Biosprint 96 DNA Blood Kit (cat. no. 940057) at the Laboratories of Analytical Biology (LAB) of the National Museum of Natural History, Smithsonian Institution (Washington, DC, USA). Specimens were barcoded using cytochrome oxidase I (COI) and additional molecular markers (mitochondrial ribosomal 16S, and nuclear-encoded ribosomal 18S and 28S) generated for phylogenetic reconstruction. The primers and PCR conditions used for obtaining 16S, 18S, and 28S were described in [47]. For COI barcoding the primers described in [48], were used for PCR (94°C for 5 min, 30 cycles of 94°C for 1 min, 50°C for 30 s, 72°C for 2.5 min, followed by a final extension step of 72°C for 5 min) and cycle sequencing.

PCRs were performed in 10 μl reactions containing 0.5 units Biolase DNA polymerase (Bioline USA Inc., Taunton, MA), 0.3 mM of each primer, 0.5 mM dNTPs (Bioline), 1.5 mM magnesium chloride, 2.5x Bovine serum albumin (BSA) (New England BioLabs Inc., Ipswich, MA), and 1x Buffer, 1 μl template DNA, and DNAase-free H_2_0 to bring the volume to 10 μl. 3 μl of a 1 in 5 dilution of ExoSAP-IT (Affymetrix, USB Products) was added to each PCR reaction, followed by incubation at 37°C for 30 min followed by 80°C for 20 min. 1 μl of the ExoSAP-IT purified PCR product was used in cycle sequencing reaction with Big Dye Terminator (v. 3.1; ThermoFisher Scientific, Waltham, MA), followed by Sephadex G-50 Fine (GE Healthcare Life Sciences, Pittsburgh, PA) clean-up. Purified sequencing reactions were then analysed on an Applied Biosystems 3130xl Genetic Analyzer or Applied Biosystems 3730xl DNA Analyzer. Sequences were assembled in Geneious (v. 9.05; Biomatters Limited, NZ) and their cnidarian origin verified by BLAST searches against the National Center for Biotechnology Information's GenBank database (http://www.ncbi.nlm.nih.gov/genbank/).

Sequences were aligned using MAFFT (v. 7.205; [49]) with default settings. The edges of the COI alignment were trimmed to remove gaps at the ends of the alignments. Kimura 2-parameter distances were calculated from this alignment with the R package APE (v. 3.5; [50]) to estimate the genetic differentiation among sampling sites.

A concatenated matrix for 16S, 18S, and 28S was constructed in Mesquite (v. 3.1; [51]) using a broad sampling of trachyline species (S1 Table), with the aim of inferring the relationship between subboreal and Antarctic *P*. *polaris* to each other, and their relationship to the remainder of Trachylina. Positions in the concatenated alignment suitable for phylogenetic analysis were identified using Gblocks [52] with the least stringent settings implemented in the alignment viewer Seaview (v. 4; [53]), allowing for smaller blocks, gap positions, and less strict flanking positions in the final alignment. The most appropriate model of sequence evolution for the aligned genes was inferred using jModelTest (v. 2.1.10; [54]) with default settings; the best fitting model was chosen using the Akaike Information Criterion (AIC). A Bayesian phylogenetic analysis was performed using MrBayes (v. 3.2.5; [55]). Here, MrBayes performed 4 separate runs with 8 markov-monte-carlo chains each for a maximum of 10,000,000 generations. Trees were sampled every 1,000 generations, discarding the first third of trees as burn-in. The analysis was stopped automatically when the average standard deviation of split frequencies among runs was < 0.01.

### Seafloor abundance and distribution

**Fig 2:**
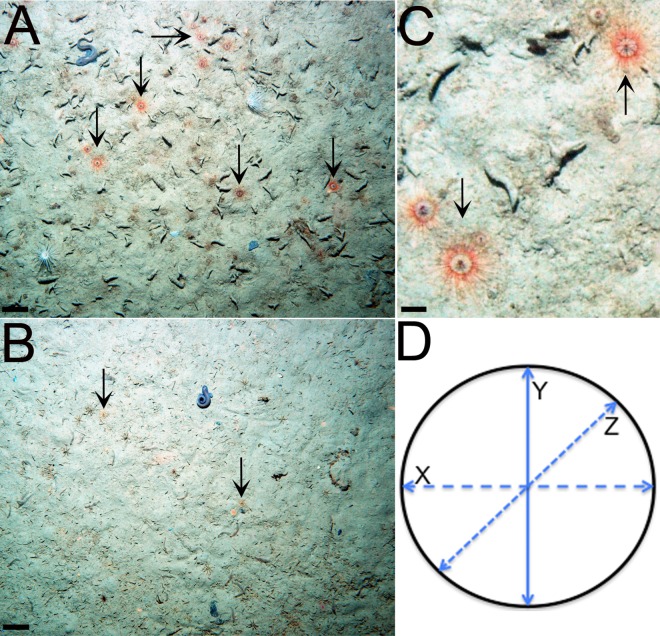
Representative images of the seafloor and the trachymedusa *Ptychogastria polaris* in Andvord and Flandres Bays. Typical view and occurrence of *P*. *polaris* in the (**A**) outer basin of Andvord Bay and (**B**) inner basin A of Flandres Bay. Scale bars are 1 cm. (**C**) Close-up view of *P*. *polaris* in the outer basin of Andvord Bay. Scale bar is 2 cm. (**D**) Bell diameter measurements taken in three horizontal directions (X, Y and Z). Note that black arrows are used to indicate the position of a representative selection of individual *P*. *polaris* in panels (**A-C**). Other *P*. *polaris* not identified by black arrows in the field of view were still counted.

Seafloor photosurveys were conducted using the Yoyo camera in Andvord, Flandres and Barilari Bays in January-February 2010 as noted in [44]. Included therein are descriptions of the environmental and substratum characteristics of the study sites [44]. In brief, two 1-km Yoyo Camera phototransects of 100 vertical images were conducted in nine fjord basins of similar water depth (436–725 m; Table 2) at two random locations in each basin, except for Barilari Bay, where one transect was completed in the outer fjord basin. Each image comprised ~3 m^2^ of the seafloor. Fifty images were randomly selected from each transect using the RANDBETWEEN function in Microsoft Excel and the abundance of *P*. *polaris* counted with the software ImageJ (ver.1.49; [56]) for a 1.8 m^2^ area in the center of each image (Fig 2A–2C). The location, on the seabed or in the water column, was also noted for each individual. Abundance is reported by fjord basin, and with distance from glacial termini (as in [44]).

**Table 2:** Seafloor photosurveys undertaken in Andvord, Flandres and Barilari Bays.

				Coordinates (Degrees Lat. and Long.)				
Site	Sampling date	Station (fjord basin)	Photo-transect (CRS #)	Transect start		Transect end		Mean depth (m)
Andvord Bay	20/01/2010	Mouth (AMTH)	1289	-64.78084	-62.87507	-64.77787	-62.86436	533
	20/01/2010	Mouth (AMTH)	1290	-64.78622	-62.87664	-64.78427	-62.88893	528
	18/01/2010	Outer (AO)	1283	-64.78213	-62.74363	-64.78044	-62.72848	551
	19/01/2010	Outer (AO)	1284	-64.78163	-62.74504	-64.78076	-62.73212	534
	20/02/2010	Middle (AM)	1337	-64.82481	-62.64829	-64.81925	-62.65908	436
	20/02/2010	Middle (AM)	1338	-64.82502	-62.65503	-64.81680	-62.65736	437
	19/01/2010	Inner (AI)	1285	-64.85837	-62.56335	-64.86339	-62.55866	523
	19/01/2010	Inner (AI)	1286	-64.65833	-62.56507	-64.86346	-62.56145	526
Flandres Bay	18/01/2010	Outer (FO)	1281	-65.00207	-63.32553	-65.00327	-63.31345	725
	18/01/2010	Outer (FO)	1282	-65.00334	-63.31835	-65.00526	-63.30604	723
	17/01/2010	Inner A (FIA)	1279	-65.05382	-63.11359	-65.05288	-63.09981	686
	17/01/2010	Inner A (FIA)	1280	-65.05316	-63.11387	-65.05795	-63.10063	672
	14/01/2010	Inner B (FIB)	1276	-65.10283	-63.15393	-65.10497	-63.13651	680
	17/01/2010	Inner B (FIB)	1278	-65.10327	-63.14998	-65.10830	-63.14260	675
Barilari Bay	27/01/2010	Outer (BO)	1300	-65.78151	-64.86280	-65.76672	-64.84957	630
	24/01/2010	Inner (BI)	1295	-65.94017	-64.63950	-65.94276	-64.65400	610
	26/01/2010	Inner (BI)	1297	-65.94334	-64.62170	-65.94826	-64.63144	610

Seafloor photosurveys for this study taken in nine basins in Andvord, Flandres and Barilari Bays during NBP10-01 (2010) from RVIB *Nathaniel B*. *Palmer*. Fjord basins are indicated as follows: AMTH = Andvord Bay mouth; AO = Andvord Bay outer; AM = Andvord Bay middle; AI = Andvord Bay inner; FO = Flandres Bay outer; FIA = Flandres Bay inner A; FIB = Flandres Bay inner B; BO = Barilari Bay outer; and BI = Barilari Bay inner.

Spatial dispersion (even versus aggregated) of *P*. *polaris* was evaluated using the variance-to-mean ratio (VMR) and Morisita’s Index of Dispersion (*I*_*d*_). Departure from randomness and the significance of both statistics were assessed by Chi-square test (χ^2^) at the 5% level [57]. Frequency distributions of trachymedusa counts were compared with the expected frequency distribution of two probability models; a Poisson (for a random distribution, variance = mean) and a negative binomial distribution (for an aggregated distribution, variance > mean). Goodness-of-fit between the observed and expected frequencies was also tested using Chi-square (χ^2^) at an alpha level of 0.05.

### Body size

Where image quality allowed, bell diameters of all *P*. *polaris* oriented flat on the seafloor within each 1.8 m^2^ area was measured in three horizontal directions (Fig 2D) using the straight line drawing tool in ImageJ (v.1.49; [56]), and a mean value calculated. Frequency size distributions of *P*. *polaris* were analysed for skewness by calculating the skewness coefficient (*g*_1_) and the standard error of skewness (*SES*). Skewness was detected by outcomes where the skewness coefficient per *SES* was > 2 or < -2 [58].

### Environmental background conditions

CTD casts to within 10 m above the seafloor were conducted in all fjord basins to measure bottom water temperature, salinity, oxygen and chlorophyll-a concentration, and to define the conditions of the *P*. *polaris* habitat. Scaled seafloor images were used to characterise substratum type (soft sediment or dropstone) underlying epibenthic trachymedusae. In an effort to develop a standardised approach, only dropstones > 3 cm x 3 cm in maximum perpendicular dimensions were considered. The frequency of *P*. *polaris* directly on dropstones was quantified.

## Results

### Morphology taxonomy and molecular systematics

**Fig 3:**
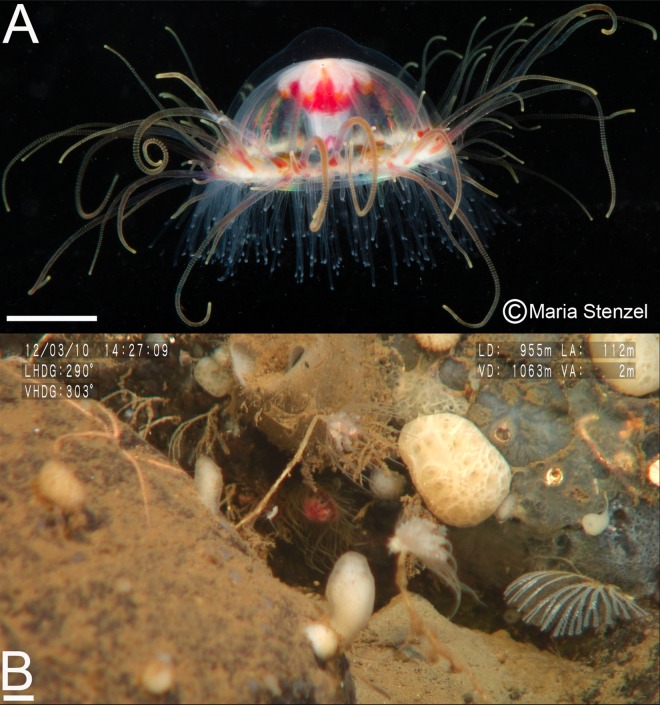
Morphological and molecular representations of *Ptychogastria polaris*. **(A**) Photograph of a live *P*. *polaris*, as photographed by Maria Stenzel, Photographer, collected by megacore in the outer basin of Andvord Bay, WAP (64.77934' S, 62.72885' W), 556 m, 22 February 2010 (Cruise NBP10-01, CRS1339). Scale bar is 1 cm. Reprinted from http://mariastenzel.photoshelter.com under a CC BY license, with permission from Maria Stenzel, original copyright 2010. **(B)** *In situ* photograph of the subboreal specimen of *P*. *polaris* collected off Okushiri Island, Sea of Japan (42.30216' N, 139.4744' E), 1062 m, 10 March 2012 (Cruise KR12-07, Dive no. 7K#0549). Scale bar is 1 cm.

Kramp synonymized *Ptychogastria polaris* and *P*. *opposita* through comparisons between net-caught specimens, which are usually damaged [33]. The present material from the Antarctic Peninsula and Japan Sea, collected by megacore and submersible, was in pristine condition (Fig 3). Because Kramp may have missed some characters due to the state of his specimens, we conducted detailed morphological comparisons between our Japan Sea and Antarctic material. Apart from the gonads being more rugose in the specimens collected in 2012 in the Japan Sea, no other differences were apparent, confirming our Antarctic trachymedusae to be *P*. *polaris* based on morphological characters and agreeing with Kramp's assertion that the two species are likely synonymous. However, COI sequences generated for this study show a large degree of differentiation between the Japan Sea and the Antarctic Peninsula with some 27% pairwise dissimilarity, while COI sequences are almost invariable within each sampled location (Table 3).

**Table 3:** Pairwise Kimura-2 parameter distances in % calculated for mitochondrial cytochrome oxidase (COI) from *Ptychogastria polaris* specimens.

	AP (KY072784)	AP (KY072785)	AP (KY072786)	JS (KY072787)	JS (KY072788)
**AP** (KY072784)	-				
**AP** (KY072785)	0.0	-			
**AP** (KY072786)	0.0	0.2	-		
**JS** (KY072787)	26.6	26.6	26.8	-	
**JS** (KY072788)	26.6	26.6	26.8	0.0	-
**JS** (KY072789)	26.5	26.5	26.8	0.2	0.2

Pairwise Kimura-2 parameter distances in % calculated for mitochondrial cytochrome oxidase (COI) from *Ptychogastria polaris* specimens collected in the Japan Sea (JS) and Antarctic Peninsula (AP). Genbank accession numbers are provided.

**Fig 4:**
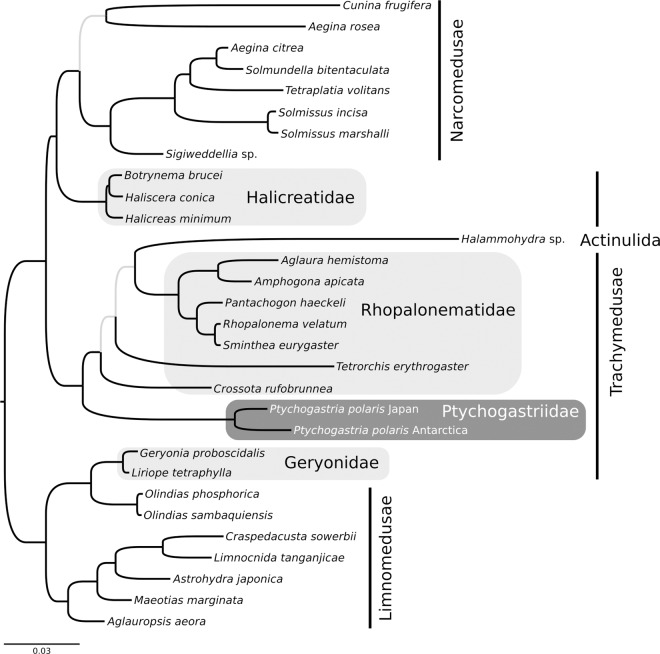
Bayesian phylogenetic hypothesis of Trachylina, including *Ptychogastria polaris*. The phylogenetic tree was rooted on Limnomedusae following [47]. Branches in black indicate a posterior probability ≥ 0.95 while grey branches represent a posterior probability < 0.95. The families of Trachymedusae are highlighted in grey. Note that the *P*. *polaris* specimen from Antarctica is lacking a 16S sequence.

Whether or not the specimens of *P*. *polaris* examined here are truly members of the same species, they are each other's closest relative, as demonstrated by phylogenetic analysis (Fig 4). Overall the topology of the phylogeny is consistent with [47]. This earlier study found Rhopalonematidae to be the closest relative to Narcomedusae [47], while the phylogeny presented here suggests that Halicreatidae is the sister to Narcomedusae. Trachymedusae is polyphyletic and Ptychogastriidae is the sister lineage to a clade containing Rhopalonematidae plus Actinulida.

### Seafloor abundance and distribution

**Fig 5:**
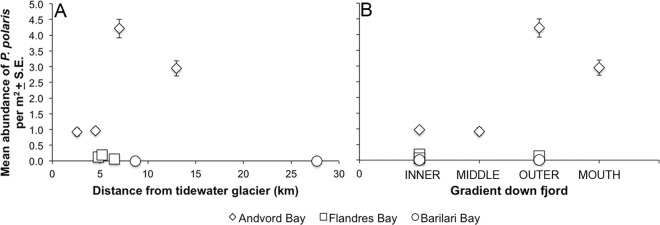
Abundance of the trachymedusa *Ptychogastria polaris* in Andvord, Flandres and Barilari Bays. Data are plotted (m^-2^) as a function of (**A**) distance to the nearest tidewater glacier, and (**B**) position in basins down fjord.

*Ptychogastria polaris* was a common component of the epibenthic community in Andvord and Flandres Bays, occurring at 7 of the 9 stations surveyed (i.e. 14 of 17 phototransects), but was absent from the inner and outer basins of Barilari Bay. A total of 1691 trachymedusae were counted in phototransects: 1626 in Andvord Bay and 65 in Flandres Bay. The highest density within a frame was 13 m^-2^, and mean abundances within basins ranged from 0.92 m^-2^ to 4.19 m^-2^ in Andvord Bay, and from 0.06 m^-2^ to 0.18 m^-2^ in Flandres Bay (Table 4; Fig 5).

**Table 4:** Spatial dispersion of *Ptychogastria polaris* in Andvord and Flandres Bays.

Phototransect CRS#	Location	Number of photographs	% photos with *P*. *polaris*	Mean no. *P*. *polaris* (m^-2^ ± SE)	VMR	*I*_*d*_	Poisson		Negative binomial		
							*n*-2	χ^2^	*n*-3	χ^2^	*k*
1289 & 1290	AMTH	100	92	2.96±0.241	3.53*	1.47*	9	143.419**	12	14.412	2.102
1283 & 1284	AO	100	94	4.19±0.294	3.72*	1.36*	10	207.455**	16	28.071**	2.775
1337 & 1338	AM	100	59	0.92±0.122	2.94*	2.17*	4	52.497**	5	2.754	0.849
1285 & 1286	AI	100	66	0.97±0.095	1.70*	1.40*	4	30.771**	4	10.871**	2.476
1281 & 1282	FO	100	19	0.12±0.027	1.06	1.30	1	0.556	1	**0.567**	3.472
1279 & 1280	FIA	100	24	0.18±0.037	1.35*	2.08*	1	3.703	1	0.251	0.942
1276 & 1278	FIB	96	9	0.06±0.019	1.11	2.13	1	0.660	1	**0.148**	0.970

Basin phototransect information and spatial distribution analysis for *P*. *polaris* observed in Andvord and Flandres Bays. Fjord basins are indicated as follows: AMTH = Andvord Bay mouth; AO = Andvord Bay outer; AM = Andvord Bay middle; AI = Andvord Bay inner; FO = Flandres Bay outer; FIA = Flandres Bay inner A; and FIB = Flandres Bay inner B. Spatial dispersion for different populations was evaluated using variance-to-mean ratio (VMR) and Morisita’s Index of Dispersion (*I*_*d*_).

* An asterisk indicates a population is significantly more aggregated than would be expected by chance for both VMR and *I*_*d*_ at the 5% level based upon Chi-square goodness of fit test. Chi-square values (χ^2^) indicate the goodness-of-fit of observed *P*. *polaris* frequencies to the expected frequency distributions of Poisson and negative binomial probability models, where the number of degrees of freedom is given by the number of frequency classes (*n*) minus 2 and 3 respectively.

** A double asterisk indicates a significant deviation from the probability distribution at the 5% level.

The exponent *k* is a parameter of the negative binomial distribution.

Note all but two cells had expected counts of at least 1.25, and 50% or fewer of the cells had expected counts of less than 5.

Bold Chi-square values indicate where one cell had an expected frequency < 1.

**Fig 6:**
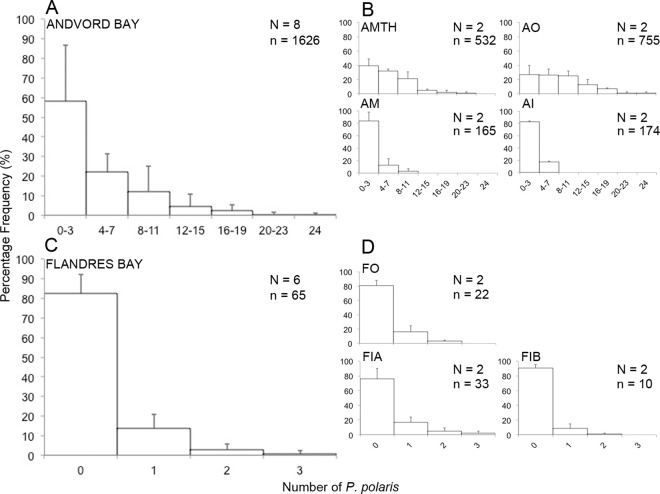
Percentage frequency distribution of the trachymedusa *Ptychogastria polaris* in Andvord and Flandres Bays. Percentage frequency distribution of *P*. *polaris* are plotted by fjord and by fjord basins using the mean + standard deviation across phototransects. (**A-B**) Andvord Bay and (**C-D**) Flandres Bay. Note that panels **A** and **C** are by fjord, and **B** and **D** are by fjord basin. Fjord basins are indicated as follows: AMTH = Andvord Bay mouth; AO = Andvord Bay outer; AM = Andvord Bay middle; AI = Andvord Bay inner; FO = Flandres Bay outer; FIA = Flandres Bay inner A; and FIB = Flandres Bay inner B. X-axes are on different scales for Andvord and Flandres Bays. N = number of phototransects and n = number of *P*. *polaris*.

There was a higher frequency of occurrence of *P*. *polaris* in Andvord Bay (commonly up to 12 individuals per frame), where maximum numbers of 23 and 24 individuals were observed in a single frame in the outer basin and at the fjord mouth respectively (Fig 6A and 6B). In contrast, in Flandres Bay, most observations of *P*. *polaris* were limited to solitary individuals, with occasionally 2–3 trachymedusae being recorded per frame (Fig 6C and 6D). Populations of *P*. *polaris* had an aggregated distribution in Andvord Bay, where the VMR and *I*_*d*_ in all fjord basins were > 1 (Table 4). Distributions of the medusae differed significantly from random (*p* < 0.05), with the negative binomial distribution providing a good fit in the middle basin of Andvord Bay and at the fjord mouth. *Ptychogastria polaris* distributions in the outer and inner basins of Andvord Bay, however, differed significantly from a negative binomial model (*p* < 0.05). An aggregated distribution was also suggested in the basins of Flandres Bay, as the VMR and *I*_*d*_ also > 1, however these distributions did not significantly differ from random (with the exception of inner basin A). Both the Poisson and negative binomial probability models were adequate descriptors for the distributions of *P*. *polaris* throughout Flandres Bay, confirming a random pattern.

Epibenthic *P*. *polaris* co-occurred with different communities in Andvord and Flandres Bays. The benthic megafaunal community in Andvord Bay was dominated by the tube-building polychaete *Amythas membranifera* Benham, 1921 and an ampeliscid amphipod (> 74% of megafaunal abundance), with *P*. *polaris* consistently being within the top 5 dominant species [44]. In contrast, Flandres Bay was characterised by the circumpolar ophiuroid, *Ophionotus victoriae* Bell, 1902 and a species of pycnogonid in one inner basin (Flandres inner basin B), with the polychaete *Pionosyllis kerguelensis* McIntosh, 1885 replacing the ophiuroid as co-dominant in the other Flandres Bay basins [44]. *Ptychogastria polaris* was among the five most numerous megabenthic species in inner basin A of Flandres Bay and among the top 11 species in the other fjord basins.

A trachymedusa similar in appearance to the epibenthic morphotype, and presumably the same species, occurred in the water column in all three fjords within a couple of meters of the seafloor, albeit in smaller numbers (468 individuals), constituting 45%, 22% and 0.5% of total demersal nekton (*Euphausia superba* Dana, 1850, species of mysid and chaetognath, and two species of pelagic medusa) in Andvord, Flandres and Barilari Bays, respectively [44]. This medusa was most abundant in the water column in Andvord Bay and at the fjord mouth. Mean densities within basins ranged from 0.26 to 0.99 m^-2^ in Andvord Bay, 0.01 to 0.16 m^-2^ in Flandres Bay and 0 to 0.02 m^-2^ in Barilari Bay. We noted two additional species of pelagic medusa in the fjords: *Benthodcodon hyalinus* Larson & Harbison, 1990 (a total of 79 individuals noted from all three fjords), and *Sigiweddellia* sp (3 individuals limited to Andvord and Flandres Bays). Both of these species lack a documented epibenthic counterpart and occurred much less frequently than *P*. *polaris*

### Body size

**Fig 7:**
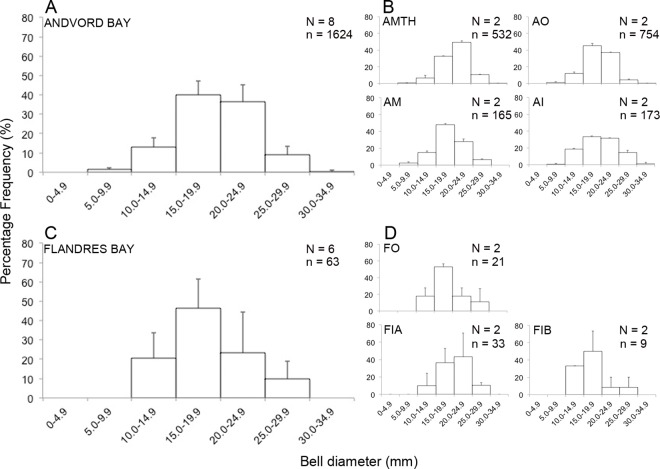
Percentage size frequency distribution of bell diameter for the trachymedusa *Ptychogastria polaris* in Andvord and Flandres Bays. Percentage *of P*. *polaris* in each size category (mm) are plotted by fjord and by fjord basins using the mean + standard deviation across phototransects. (**A-B**) Andvord Bay and (**C-D**) Flandres Bay. Note that panels **A** and **C** are by fjord, and **B** and **D** are by fjord basin. Fjord basins are indicated as follows: AMTH = Andvord Bay mouth; AO = Andvord Bay outer; AM = Andvord Bay middle; AI = Andvord Bay inner; FO = Flandres Bay outer; FIA = Flandres Bay inner A; and FIB = Flandres Bay inner B. N = number of phototransects and n = number of *P*. *polaris*.

A total of 1624 measurable *P*. *polaris* were recorded in Andvord Bay, with an average bell diameter of 20 mm ± 4 SD and a range between 8 to 32 mm. An asymmetric, negatively skewed distribution was observed at the fjord level in Andvord Bay, and at the mouth of the fjord at the basin level (Table 5), with most trachymedusae in the intermediate size classes (15–20 and 20–25 mm; Fig 7A and 7B). The sixty-three measurable *P*. *polaris* in Flandres Bay averaged 18 mm ± 4 SD in diameter, with a range of 11 to 28 mm. Skewness coefficients in *P*. *polaris* indicated that the Flandres Bay size distributions were not significantly different from unimodal/symmetric (Table 5; Fig 7C and 7D). Similarly to Andvord Bay, most individuals were between 15 and 25 mm in diameter, however the smallest size class (5–10 mm) was absent from Flandres Bay.

**Table 5:** Skewness characteristics of *Ptychogastria polaris* size frequency distributions observed in Andvord and Flandres Bays.

Site	*g*_1_	*SES*	Sig. (> 2)
Andvord Bay	-0.17	0.06	-2.75
AMTH	-0.41	0.11	-3.83
AO	-0.17	0.09	-1.93
AM	0.05	0.19	0.25
AI	0.03	0.18	0.15
Flandres Bay	0.37	0.30	1.23
FO	0.51	0.41	1.25
FIA	0.76	0.72	1.06
FIB	0.39	0.50	0.79

Skewness coefficient (*g*_1_) and the standard error of skewness (*SES*) were calculated. Skewness is detected if the absolute value of *g*_1_/*SES* is > 2 or < -2 (Sig. > 2). If the absolute value of *g*_1_/*SES* is lower than minus two (greater than plus two) then this suggests the population is negatively (positively) skewed. Fjord basins are indicated as follows: AMTH = Andvord Bay mouth; AO = Andvord Bay outer; AM = Andvord Bay middle; AI = Andvord Bay inner; FO = Flandres Bay outer; FIA = Flandres Bay inner A; and FIB = Flandres Bay inner B.

### Environmental background conditions

The highest basin mean abundance of *P*. *polaris*, 4.19 m^-2^ ± 0.29 SE, was recorded in the outer basin of Andvord Bay at ~535–550 m. *Ptychogastria polaris* was also common (basin mean abundance 2.96 m^-2^ ± 0.24 SE) at the mouth of the fjord at a depth of ~530 m. Lower abundances were recorded at shallower sites (~ 430–520 m), where ~ 1 m^-2^ was commonly encountered, and at depths > 600 m (0.06 to 0.17 m^-2^).

*Ptychogastria polaris* occurred in bottom water temperatures ranging from ^-^0.02 to^+^0.98°C. Salinity varied little across sampling sites (34.50–34.53), and oxygen and chlorophyll-a concentrations had mean values of 5.16 ml/l ± 0.38 SD (4.80–5.71 ml/l) and 0.03 μg/l ± 0.08 (0–0.25 μg/l), respectively.

Fjord basin floors were characterised by soft-sediments (e.g. dropstones covered only 1.2% of the seafloor in Andvord Bay, A. Ziegler unpublished data). Most trachymedusae could be classified as epibenthic with ~ 80% in contact with, or just overlying soft sediment; less than 1.5% of *P*. *polaris* were associated with dropstones, most of which were sediment-covered.

## Discussion

The trachymedusa *Ptychogastria polaris* attains high seafloor densities in both Andvord and Flandres Bays. Densities are up to 400-fold higher than reported for *P*. *polaris* in Arctic locations in NE Greenland (0.01 and 0.91 ind per m^2^; [25]), and the Barents Sea (0.01–0.52 ind per m^2^; [25]; 0.41–0.76 ind m^-2^, [26]), and exceed numbers of specimens (1–6 individuals) collected by dredge from two widely separated areas in the Southern Ocean (near Gauss Station [35], and the South Shetland Islands [33]). Such pronounced densities may result from higher benthic productivity in the fjords, potentially via higher detrital inputs from sustained phytoplankton blooms, macroalgae cascading down fjord walls, and/ or horizontal nutrient subsidies (e.g. carcasses and faeces from migrating Antarctic krill, *Euphausia superba* and baleen whales [44]). Concentrations of medusae near the seafloor of submarine canyons have also been associated with sizeable accumulations of organic and inorganic debris in the form of sediment and detritus [59]. These bathymetric features may resemble subpolar fjords in depth, sediment characteristics, oceanographic processes (e.g. enhanced primary production and the trapping of eddies) and thus in the ability to concentrate migrating nekton; such features appear to provide suitable habitats for midwater and benthopelagic medusae, including *P*. *asteroides*, the Mediterranean counterpart of *P*. *polaris* [16, 17, 59, 60]. The ecological and oceanographic features that facilitate the concentration of medusan fauna in submarine canyons and subpolar fjords may therefore explain why we observe a relatively higher richness and abundance of benthopelagic medusae in these unique ocean habitats compared to the generic deep sea. However, owed to inadequate sampling efforts, via the tools deployed and the frequency of collection (summarised in [10]), bipolar taxa such as *P*. *polaris* appear underrepresented, but may in fact be cosmopolitan in distribution.

*Ptychogastria polaris* was widespread throughout the sediment-floored basins of Andvord and Flandres Bays. The basin and fjord-scale distribution patterns vary, however, with generally higher seafloor abundances towards the outer reaches of the fjords, and a higher abundance in Andvord Bay, where *P*. *polaris* exhibited an aggregated distribution. Small-scale jellyfish aggregations are commonly reported from coastal waters, but typically for the pelagic medusae [61, 62, 63, 64]. Although “swarms” of a similar magnitude are not recognized at the seafloor, other epibenthic medusae, in addition to *P*. *polaris*, are reported to accumulate in small, aggregated populations that are densely packed, such as medusae of the scyphozoan upside-down jellyfish of the genus *Cassiopea* Péron & Lesueur, 1810 and benthic staurozoans [64]. In contrast, the occurrence of solitary individuals per 1.8 m^2^ and low-density populations with apparently random dispersion patterns, were observed in Flandres Bay, which is consistent with patterns reported for *P*. *polaris* in the Arctic [25, 26].

Patches of medusae form as a consequence of the physical, chemical and biological interactions of jellyfish with the marine environment (reviewed by [11]). Topography and environmental parameters such as rates of sedimentation, carbon input, or fjord circulation patterns may therefore influence the distribution of the trachymedusa within and between the fjords. There are, however, only limited measurements of sedimentation rates in Andvord and Flandres Bays, with rates in inner basins of both fjords of approximately 3–5 mm yr^-1^ [65]. Sedimentation rates are likely to decline down fjord but are poorly constrained [66, 67], and accurate measurements of neither key inputs nor oceanographic parameters are available in the published literature. Available observations suggest the fjords are quiescent systems, with weak melt-water input compared with most Arctic fjords ([68, 69]; C. Smith pers. observ.), and elevated current velocities at the fjord mouths. Localised regions of turbulent mixing over sills are also anticipated in Andvord and Flandres Bays. The complex interplay between shelf processes, wind, ice-ocean interactions and tidal inputs limits the predictability of spatial and temporal variability in water column currents that could influence the distribution patterns observed for *P*. *polaris*.

*Ptychogastria polaris* is distributed amongst a diversity of epibenthic megafauna in the WAP fjords, often dominating the community composition alongside species of echinoderms, polychaetes and crustaceans [44]. Trachymedusae of this species also co-occur with rich epibenthic communities that are often dominated by echinoderms (e.g. *Ophiura robusta* Ayres, 1854 and *Strongylocentrotus pallidus* Sars G.O., 1872; [26]) in the Arctic at shelf depths off Northeast Greenland and in the Barents Sea [70, 71]. Most *P*. *polaris* recorded in the fjords were observed at or hovering a few centimetres above the seafloor, which is consistent with laboratory and field reports describing a lifestyle that includes supra- and epibenthic living positions [2, 25, 26, 33, 42, 59]. Some medusae, the morphology of which would otherwise be interpreted to have a wholly pelagic lifestyle, have also been suggested to feed in a benthic mode (e.g. [72]), while other medusae are well known to have a benthic/benthopelagic ecology (e.g. medusae within the genera Cladonema, Eleutheria and Staurocladia [2, 73, 74]). Other indicators of a benthic association in *P*. *polaris* include anatomical and functional adaptations to the benthic boundary layer, including adhesive tentacle tips, allowing attachment to hard substrata [2, 32, 42], and a relatively low number (~ 40–50) of spawned eggs that adhere to the sediment-water surface [2].

Despite evidence from both the Arctic and boreal waters of a demersal existence ([2, 25, 26, 33, 42, 59], this study), *P*. *polaris* along the WAP was also observed in the water column, albeit at smaller densities, in all three fjords including Barilari Bay. The trachymedusa is known to undertake short swimming excursions (~ 15 s [2]; D. Stübing unpublished data, referred to in [25]), often in response to disturbance (e.g. caused by contact with swimming euphausids as described in [2]). However, the relatively high frequencies of occurrence in the water column recorded here have not been reported previously in the published literature suggesting this Antarctic trachymedusa may behave differently from its Arctic and boreal counterparts.

Periodic swimming has been reported for other benthic medusae, including *P*. *asteroides*, the Mediterranean counterpart of *P*. *polaris* [59] and several members of the Limnomedusae [75, 76, 77]. In contrast to the brief excursions reported for *P*. *polaris*, *P*. *asteroides* spends more time in the water column [59]. These swimming periods have been associated with perturbations in deep-water flows and the associated sediments and detritus commonplace in Mediterranean submarine canyons, to which this trachymedusa is endemic [59]. The nocturnal emergence behaviour observed in other species has been attributed to the light-inhibited and diel-feeding activities of the Limnomedusae. Oceanographic conditions in the WAP fjords are not well constrained, however localised regions of turbulent mixing over sills, distinct circulation processes and sediment inputs unique to the fjords could explain the intermittent swimming behaviour of *P*. *polaris*. Although not empirically tested, feeding opportunities are also likely to drive the behavioral adaptations of the trachymedusa in WAP fjords.

The size range of *P*. *polaris* (8 to 32 mm in bell diameter) in the WAP fjords is similar to that in Northeast Greenland (7 and 29 mm, median 14 mm [25]), the Barents Sea (5 and 21 mm, median 13 mm [25]; mean 21 mm [26]), and in the historical literature (maximum values of 18 to 24 mm, [78]). The distributions of bell diameter were mostly unimodal, with the only difference being a higher proportion of intermediately-sized individuals towards the outer basin and mouth of Andvord Bay, suggesting a single pulse or seasonal recruitment event, and larger, mature trachymedusae generally account for most *P*. *polaris* in the WAP fjords. Differences in the proportion of medusae distributed amongst the various size spectra indicate that the timing of recruitment and/or growth rates of *P*. *polaris* may differ between Andvord and Flandres Bays. The fact that none of the *P*. *polaris* observed in Flandres Bay corresponded to the smallest size class sampled suggests recruitment is lower compared to, and/ or out of phase with, Andvord Bay, which is concert with the lower seafloor and water column densities recorded in this fjord.

In the WAP fjords, *Ptychogastria polaris* occurred in seawater temperatures (^-^0.02°C to^+^0.98°C) similar to those in in Northeast Greenland and the Barents Sea (^-^1.6°C to ^+^2.1°C; [25, 26]), confirming *P*. *polaris* as a cold-water species, originally as asserted by [32] and supported by [41]. Although most historical observations report this species as attached to hard substrata in Arctic and boreal waters [2, 32, 42] (Fig 3B and 3D. Lindsay unpublished data), the WAP fjord floors are characterised by soft-sediment, with only 1.5% of *P*. *polaris* individuals associated with dropstones. Associations with silty sand and finer sediment affinities have since been confirmed in the Arctic [25, 26]. *Ptychogastria polaris* therefore appears to have context-dependent habitat preferences.

This investigation provides the first phylogenetic analysis containing *Ptychogastria*, and more broadly the family Ptychogastriidae. The morphological taxonomy confirms the WAP fjord trachymedusa as synonymous with the Arctic *P*. *polaris* described by [31] and the Antarctic *P*. *opposita* designated by [35]. Thus, *Ptychogastria* is a monotypic bipolar genus with two species (*P*. *asteroides* from the Mediterranean and *P*. *polaris* from the northern and southern high latitudes). This discovery revises the designation of *Ptychogastria* as a genus represented by three species [45], and agrees with the conclusions of Kramp, that the Arctic and Antarctic forms are conspecific [33, 34], and that *P*. *polaris* is one of 23 bipolar species belonging to the Medusozoa [45]. However, in contrast to the lack of morphological differences, genetic differences are large, at least for the fast evolving barcoding marker COI. *Ptychogastria polaris* appears to be most closely related to the pelagic medusae of the family Rhopalonematidae (plus the sand-interstitial dwelling Actinulida), furthering our understanding of trachymedusan classification and evolution. Future studies are needed to assess the genetic structure and demographic connectivity of widely distributed populations of *P*. *polaris*. In this context, the high differentiation uncovered in the barcoding molecule COI is of interest. The differentiation observed here, being larger than 25% in pairwise sequence comparisons, is at odds with the lack of distinguishing morphological characters. This differentiation is far greater than has been reported for hydrozoan species (~4%), and is more in line with previously described inter-specific differences (~20%; [79]). Additional sampling for *P*. *polaris* on broader geographic scales should determine whether this species contains multiple morphologically cryptic species or shows an unusually high degree of sequence variation between the opposite, widely separated ends of its distributional range.

The relatively high densities of *P*. *polaris* in Andvord and Flandres Bays suggest that live or dead *P*. *polaris* may link pelagic and benthic food-webs within WAP subpolar fjords. Zooplankton, including gelatinous taxa, can connect pelagic and benthic subsystems in a variety of ways, contributing prey and fecal material, undertaking vertical migrations and through dispersing life history stages [9, 80, 81]. For example, the provision of fecal pellets by extremely abundant krill and salps and their direct consumption by benthic filter feeders enhances the efficiency of exchange between the pelagic and benthic subsystems in the Antarctic [80]. Epibenthic medusae that are present in equally large numbers, such as *P*. *polaris*, and that make excursions into the benthopelagic layer may also play an important ecological role as predators of epibenthic organisms, consuming detritivorous zooplankton that are responsible for much of the secondary production within the benthic boundary layer [12, 18, 73, 82]. In addition, jelly falls provide food inputs to the seafloor [83, 84]. For these reasons, high densities of epibenthic and benthopelagic medusae could yield food subsidies to fjord floors and integrate the trophic ecology of the WAP deep-fjord benthos, influencing processes of energy transfer between the pelagic and benthic components of the marine environment. Further research into trophic linkages and food-web dynamics in the WAP fjords should resolve the extent to which *P*. *polaris* subsidizes the benthos, and influences benthic community composition and functioning.

## Supporting Information

S1 Table Genbank accession numbers. Genbank accession numbers for large subunit (LSU), small subunit (SSU) and 16S sequences that were used in reconstructing the phylogenetic hypothesis in the present contribution. Bold accession numbers indicate sequences generated for this study and dashes are missing data.
(PDF)
