# Supplementary material for: High Abundance of the Epibenthic Trachymedusa Ptychogastria polaris Allman, 1878 (Hydrozoa, Trachylina) in Subpolar Fjords along the West Antarctic Peninsula
Source: PLoS One. 2017 Jan 4;12(1):e0168648. doi: 10.1371/journal.pone.0168648 (PMC5214585; doi:10.1371/journal.pone.0168648)
Supplement: S1 Table — Genbank accession numbers for large subunit (LSU), small subunit (SSU) and 16S sequences that were used in reconstructing the phylogenetic hypothesis in the present contribution. Bold accession numbers indicate sequences generated for this study and dashes are missing data. (PDF) [file pone.0168648.s001.pdf]

S1 Table. Genbank accession numbers for sequences that were used in reconstructing the phylogenetic hypothesis in the present contribution. Bold accession numbers indicate sequences generated for this study and dashes are missing data.

Taxon		LSU	SSU	16S
**Trachymedusae**	*Aglaura hemistoma*	**KY077268**	EU247820	EU293984
	Aglauropsis aeora	AY920793	AY920754	EU293973
	Amphogona apicata	**KY077269**	**KY077279**	**KY077288**
	Botrynema brucei	EU247798	EU247822	EU293982
	Crossota rufobrunnea	EU247799	EU247823	EU293987
	Geryonia proboscidalis	EU247807	EU247816	EU293979
	Halicreas minimum	**KY077270**	**KY077280**	**KY077289**
	Haliscera conica	EU247797	EU247825	EU293981
	Liriope tetraphylla	**KY077271**	**KY077281**	**KY077290**
	Maeotias marginata	EU247810	AF358056	AY512508
	Pantachogon haeckeli	AY920792	AF358062	**KY077291**
	Ptychogastria polaris	**KY077272**	**KY077282**	**KY077292**
	Ptychogastria polaris	**KY077273**	**KY077283**	-
	Rhopalonema velatum	EU247804	EU247819	EU293992
	Sminthea eurygaster	-	**KY077284**	**KY077293**
	Terorchis erythrogaster	**KY077274**	**KY077285**	EU293995
**Actinulida**	*Halammohydra*sp.	EU301623	EU2301622	EU293991
**Limnomedusae**	*Astrohydra japonica*	AY920794	**KY077286**	EU293975
	Craspedacusta sowerbii	**KY083050**	**KY077287**	**KY077294**
	Limnocnida tanganjicae	AY920795	AY920755	**KY077295**
	Olindias phosphorica	EU24708	AY920753	EU293978
	Olindias sambaquiensis	**KY077275**	EU247814	EU293977
**Narcomedusae**	*Aegina citrea*	AY920789	AF358058	EU293997
	Aegina rosea	-	EU247813	-
	Cunina frugifera	**KY077276**	AF358059	-
	*Sigiweddellia*sp.	**KY077277**	**KY007607**	**KY007593**
	Solmissus incisa	**KY077278**	**KY007609**	**KY007596**
	Solmissus marshalli	AY920790	AF358060	EU294001
	Solmundella bitentaculata	EU247795	EU247812	EU293998
	Tetraplatia volitans	DQ002502	DQ002501	EU293999
